# Community-based prevalence of typhoid fever, typhus, brucellosis and malaria among symptomatic individuals in Afar Region, Ethiopia

**DOI:** 10.1371/journal.pntd.0006749

**Published:** 2018-10-04

**Authors:** Biruk Zerfu, Girmay Medhin, Gezahegne Mamo, Gezahegn Getahun, Rea Tschopp, Mengistu Legesse

**Affiliations:** 1 Department of Medical Laboratory Sciences, College of Health Sciences, Addis Ababa University, Addis Ababa, Ethiopia; 2 Microbiology Research Unit, Aklilu Lemma Institute of Pathobiology, Addis Ababa University, Addis Ababa, Ethiopia; 3 Department of Veterinary Microbiology, Immunology and Public Health, College of Veterinary Medicine and Agriculture, Addis Ababa University, Bishoftu, Ethiopia; 4 Melka Werer Health Center, Amibara district, Melka Were, Ethiopia; 5 Armauer Hansen Research Institute, Addis Ababa, Ethiopia; 6 Swiss TPH, Basel, Switzerland; Mahidol Univ, Fac Trop Med, THAILAND

## Abstract

**Background:**

In sub-Saharan Africa, where there is the scarcity of proper diagnostic tools, febrile illness related symptoms are often misdiagnosed as malaria. Information on causative agents of febrile illness related symptoms among pastoral communities in Ethiopia have rarely been described.

**Methods:**

In this a community based cross-sectional survey, we assessed the prevalence of typhoid fever, typhus, brucellosis and malaria among individuals with a set of given symptoms in Amibara district, Afar Region, Ethiopia. Blood samples were collected from 650 study participants, and examined by Widal and Weilfelix direct card agglutination test (DCAT) as well as test tube based titration test for *Salmonella enterica* serotype Typhi (*S*. Typhi) and *Rickettsia* infections. Rose Bengal Plate Test (RBPT) and Complement Fixation Test (CFT) were used to screen *Brucella i*nfection. Thin and thick blood smears were used to diagnosis malaria.

**Results:**

Out of 630 sera screened by DCAT, 83 (13.2%) were reactive to H and/or O antigens for *S*. Typhi infection. Among these, 46 (55.4%) were reactive by the titration test at the cut off value ≥ 1:80. The combined sero-prevalence for *S*. Typhi by the two tests was 7.3% (46/630). The seroprevalence for *Rickettsia* infection was 26.2% (165/630) by DCAT and 53.3% (88/165) by the titration test at the cut off value ≥ 1:80. The combined sero-prevalence for *Rickettsia* infection by the two tests was 14.0% (88/630). The sero-prevalence for *Brucella* infection was 12.7% (80/630) by RBPT, of which 28/80 (35%) were positive by CFT. The combined sero-prevalence for *Brucella* infection by the two tests was 4.4% (28/630). Out 650 suspected individuals for malaria, 16 (2.5%) were found positive for *P*. *falciparum* infection.

**Conclusion:**

In this study, typhoid fever, typhus, brucellosis and malaria were observed among symptomatic individuals. The study also highlighted that brucellosis cases can be misdiagnosed as malaria or other disease based solely on clinical diagnosis. Therefore, efforts are needed to improve disease awareness and laboratory services for the diagnosis of brucellosis and other zoonotic diseases to identify other causes of febrile illness in this pastoral setting.

## Author summary

Many diseases such as typhoid fever, typhus, brucellosis and malaria show common symptoms such as fever, headache, joint pain and back pain. Hence, in countries where there is a problem of appropriate laboratory based diagnostic tools, health workers cannot properly diagnose these diseases and provide appropriate treatment. A community- based studies of the causative agents of the above mentioned illness would provide important information for health workers about some of the common causative agents in that particular area. In this study, we assessed the prevalence of typhoid fever, typhus, brucellosis and malaria among individuals who were complaining illnesses such fever, headache, joint pain and back pain in the pastoral of the Amibara district, Afar Region, Ethiopia. Among 650 individuals who were complaining symptoms, 46 (7.3%), 88 (14.0%), 28 (4.4%) and 16 (2.5%) were diagnosed for typhoid fever, typhus, brucellosis and malaria in that order. However, for the majority of the participants (75.4%), the cause of their illness remained unknown, and further investigations on the causative agents of febrile illness related symptoms is important in the present study area.

## Introduction

Sub-Saharan Africa is plagued by a myriad of infectious diseases posing significant public health and economic challenges. In addition, the often non-specific clinical signs of these diseases and the scarcity of proper diagnostic tools are the major challenges for health professionals in properly diagnosing and treating adequately patients [1, 2]. Studies showed that symptoms such as fever, headache, joint pain and back pain are often misdiagnosed as malaria, especially until the introduction of rapid diagnostics for malaria though these symptoms are not only specific to malaria [3–5].

Many studies have shown that diseases such as typhoid fever, rickettsioses, brucellosis, Q fever, and leptospirosis are the leading causes for febrile illness with symptoms such as fever, headache, joint pain and back pain [6–9]. For instance, typhoid fever due to *S*. Typhi has been reported as the leading cause of over 21 million febrile cases and over 200,000 deaths each year in many low- and middle-income countries [7–9].

Brucellosis has been considered as an important zoonotic disease worldwide and is responsible for big economic losses as it causes abortion in livestock [10, 11]. It also causes a considerable human morbidity and spontaneous abortion among pregnant women in endemic areas [12–15].

Although many malarious countries including Ethiopia are scaling up malaria intervention programs towards elimination, the disease remains one of the worst health problems with an estimated 216 million cases and 445 000 deaths globally in 2016, while most of the cases and deaths occurred in African [16]. Hence, in low- and middle-income countries where there is a shortage of effective routine diagnostic tools to identify a wide range of infectious diseases that manifest similar symptoms and where there is also a low awareness among community members and health professionals about the common causative agents of such illnesses, a community-based approach epidemiological survey would help health professionals to improve clinical diagnosis and provide appropriate treatment [1, 2].

In Ethiopia, there have been few health facilities based studies to determine the prevalence of typhoid fever, typhus, malaria and brucellosis among individuals presented with febrile illness related symptoms [17–19]. Communities based epidemiological data on the causative agents of common febrile illness related symptoms is generally lacking in the pastoralists areas due to the remoteness of sites and pastoralists way of life. Moreover, in the present study area, health professionals had no clear information on the magnitude of brucellosis, and its clinical based diagnosis might not be even considered. Hence, we assessed the prevalence of typhoid fever, typhus, brucellosis and malaria in individuals who were complaining of symptoms such as fever, headache, joint pain and back pain in the pastoral community of the Amibara district, Afar Region, Ethiopia.

## Material and methods

### Study area and population

The study was conducted in the pastoral community of Amibara district in the Afar Region of Ethiopia, around 260 km from Addis Ababa. The majority of the study population are pastoralists, depending on livestock for their livelihoods, while some started to practice agro-pastoralism and growing crops along river Awash. The study area and the population has been previously described in detail [20, 21].

### Study design and sample size estimation

A community based cross-sectional survey was carried out between September and December 2016 to determine the prevalence of typhoid fever, typhus, brucellosis and malaria among individuals who were complaining of a range of symptoms. The result of previous health facilities based sero-prevalence of brucellosis (34%) in other pastoral community areas of Ethiopia was used to estimate the sample size [17] for the study on brucellosis. Using this information, a sample size of 380 individuals (95% confidence level, 5% degree of accuracy and 10% compensation for refusal of blood sample) was initially considered. According to the information from Melka Worer health center 50% of patients with symptoms like fever, headache and joint pain often diagnosed as positive for typhoid, typhus or malaria. Based on this supplementary information, sample size was increased to 422 individuals (95% confidence level, 5% degree of accuracy and 10% compensation for refusal of blood sample). However, during the survey period all eligible individuals who came for diagnosis were considered and the sample size was increased to 650.

### Study procedure and study participants

In this study, six accessible pastoral kebeles of the district were conveniently selected, and a house-to-house survey of all households in the selected kebeles was conducted by community health workers under the supervision of the research team. The heads of the households (husband or wife) or individuals over 18 years were asked if there was any family member (age ≥ 2 years) who manifested symptoms such as fever, fatigue, headache, joint pain, and back pain for short or long periods of time. The individuals were asked to come to the nearest health post, and they were interviewed in their local language (Afar language) using a structured questionnaire that captured common signs/symptoms they felt, onset of illness, treatment sought, and information on socio-demographic characteristics of the individuals. Body temperature was also recorded using a digital thermometer. All individuals equal or older than 2 years, who reported the above symptoms, came for examination and willing to provide blood, and gave informed consent and/or assents were included in the study.

### Blood sample collection and examination

Three ml venous blood sample was collected into plain vacutainer test tube and transported to Melka Werer Health Center. Serum was separated and tested for typhoid fever, typhus and malaria on the same days. The remaining serum was stored at -20°C until transported to the laboratory of Aklilu Lemma Institute of Pathobiology, Addis Ababa University and tested for brucellosis and also tested for typhoid and typhus by test tube based titration method. Anemic individuals and pregnant women were included in the malaria study and provided only finger prick blood sample.

Widal and Weilfelix direct card agglutination tests (DCAT) were used for the serological screening of *S*. Typhi and *Rickettsia* infections, respectively following the manufacturer’s instructions (Rapid Labs Ltd, Hall Farm Business Centre, UK), and as previously described [22, 23]. A test tube based titration test was performed for all samples that were found to be reactive by the DCAT and for other 25 randomly selected samples which were found non-reactive as previously described [24]. Rose Bengal Plate Test (RBPT) was used to screen for *Brucella* infection as previously described [25]. All sera which tested positive by the RBPT and other randomly selected 68 negative samples were further tested using Complement Fixation Test (CFT) following the guidelines of OIE 2008 [26]. The guideline of Ministry of Health (MOH) was followed for the diagnosis of malaria and identification of Plasmodium species at the Health center [27].

### Data entry and analysis

Data was entered into EpiData 3.1 and analyzed with Stata/SE 11.0. Descriptive analysis was used to summarize the data in the form of frequencies and percentages of variables. Pearson chi-square test was used to evaluate the statistically significant difference in the level of prevalence of typhoid fever, typhus, brucellosis and malaria between male and female study participants and according to the reported clinical features. Bivariable and multivariable logistic regression analyses were performed to explore associations of socio-demographic characteristics of the study participants with increased odds of having higher prevalence of typhoid fever, typhus, brucellosis and malaria. P-value below 5% was considered as indicator of statistical significance.

### Ethical consideration

This study received ethical clearance from the Institutional Review Board of Aklilu Lemma Institute of Pathobiology, Addis Ababa University (ALIPB/IRB/005/2015/16)). Permission was obtained from Amibara Health Office. Participants’ information sheet which contains the objective of the study, inclusion/exclusion criteria, the required data and methods of data collection as well as informed consent document were prepared in Amharic the national language of the country. Then, the elements of participants’ information sheet initially were orally translated to the local language and described to community leaders and to each of the study participant or parent in case of children under 18 years by trained local health personnel. Informed written consent was obtained from illiterate participants and/or assent in children aged between 12 and 18 years by signing with their finger. Blood sample was collected under aseptic condition by experienced laboratory technicians. Study participants who were found positive for the investigated diseases were treated accordingly as per physician recommendation.

## Results

### Socio-demographic characteristics of the study participants

A total of 657 individuals who were complaining various symptoms such as headache, joint pain fever and back pain appeared for clinical examination. However, seven individuals were not volunteers to consent to provide blood sample and they were excluded. Out of the 650 study participants, 630 provided venous blood and 20 provided finger prick blood sample due to anemia and/or pregnancy. The participants’ age ranged from 2 to 80 years with mean age 34.25 ±17.38 years. The majority were illiterate (75.2%) and pastoralists (78.8%) (Table 1).

**Table 1:** Socio-demographic characteristics of the study participants.

Variables		Number (%)
Sex	Male	253 (38.9)
	Female	397 (61.1)
Age (years)	2–14	114 (17.5)
	15–24	59 (9.1)
	25–34	146 (22.5)
	35–44	120 (18.5)
	≥45	211 (32.5)
Religion	Muslim	612 (94.2)
	Others	38 (5.8)
Ethnicity	Afar	592 (91.1)
	Others	58 (8.9)
Education status	Illiterate	489 (75.2)
	1–12 grade attended	61(9.4)
	Non-schooled children	100 (15.4)
Occupation	Pastoralist	514 (79.1)
	agro pastoralist	67(10.3)
	Others	69 (10.6)
Marital status	Married	474 (72.9)
	Single/children	133 (20.5)
	Other	43 (6.6)

### Clinical signs/symptoms reported by the study participants

Table 2 shows the clinical signs, duration of the illness and treatment history as reported by the study participants. Headache (74.8%), joint pain (74.8%), and general malaise (24.9%) were the frequently reported symptoms. The duration of the illness reported by the participants ranged between 2 and 7300 days, with a median duration of 257 days. A total of 159 individuals (24.5%) reported that they sought treatment at various health facilities for their or their family member current illness. Among them, 68/159 (42.8%) were examined and treated for malaria, typhus or typhoid fever, while others (91 participants) reported that they and their families received a treatment though they did not get adequate information for which disease they were treated. The remaining 491(75.5%) individuals did not seek treatment because of various reasons like distance from health facility, the intermittent nature of the illness or lack of money. History of abortion was reported by 116/325 (35.7%) women and the majority (70.7%) of them didn’t know the cause of the abortion.

**Table 2:** Clinical examination, duration of illness and treatment history as reported by the study participants.

Clinical features		Number (%)
Body temperature	≥ 37.5°c	32 (5.4)
Clinical symptoms	Headache	486 (74.8)
	Joint pain	486 (74.8)
	Malaise	162 (24.9)
	Back pain	145 (22.3)
	Weakness	92 (14.1)
	Vomiting	10 (1.5)
Illness duration	Less than 8 days	249 (38.9)
	8–30 days	162 (25.3)
	Greater than 30 days	229 (35.9)
Health facility visited	No	491 (75.5)
	Yes	159 (24.5)
Reason for not visited health facility	Lack of money	101(20.7)
	Distance from health facility	59 (12.1)
	intermittent nature of the illness	118 (24.1)
	Other reasons like work load	211 (43.1)
Abortion history in women	No	209 (64.3)
	Yes	116 (35.7)
Cause of the abortion	Disease	8 (6.9)
	Accident	26 (22.4)
	Unknown	82 (70.7)

### Sero prevalence of *S*. Typhi infection

Out of 630 sera screened by the DCAT, 83 (13.2%) were reactive for *S*.Typhi infection either against flagella (H) antigen (18/83, 21.7%) or against somatic (O) antigen (41/83, 49.4%) or against both H and O antigens (24/83, 28.9%).

Among the reactive sera to O antigen, 17(19.54%), 8(9.20%), 1(1.2%) and 1(1.2%) were reactive at the titration of 1:80, 1:160, 1:320 and 1:640, respectively. Among the reactive sera to H antigen, 15 (22.73%), 9 (13.64%) and 1(1.52%) were reactive at the titration of 1:80, 1:160 and 1:320, respectively. Thus, the overall sero-prevalence of current infection with *S*. Typhi as indicated either by H and/or O antigen was considered as 7.3% (46/630) at the cut off value ≥ 1:80. The cases were more common among females than among males (17.2% vs 6.9%, X^2^ = 14.06, P<0.001) as detected by DCAT and by the titration test (9.4% vs. 4.1%, X^2^ = 6.35, P = 0.012). All the randomly selected 25 samples which were non-reactive by DCAT were also found non- reactive by the titration test.

In multivariable regression analyses, being female (AOR = 2.21, 95%CI: 1.01–4.83, P = 0.047) and duration of illness above a month (AOR = 2.70, 95%CI: 1.02–7.18, P = 0.046) were found to be associated with a high sero-positivity for *S*. Typhi infection (Table 3).

**Table 3:** Socio-demographic of the study participants and prevalence of typhoid fever.

characters		Number tested	Number (%) positive by titration	AOR(95%CI)	P value
Sex	Male	247	10 (4.1)	1	
	Female	383	36 (9.4)	2.21(1.01–4.83)	0.047
Age (years)	2–14	104	4 (3.9)	1	
	15–24	58	5 (8.6)	1.30(0.12–13.47)	0.828
	25–34	144	11 (7.6)	1.11(0.11–11.30)	0.930
	35–44	117	10 (8.6)	1.03(0.10–10.62)	0.977
	≥ 45	207	16 (7.7)	1.15(0.11–11.60)	0.906
Education	Illiterate	479	38 (7.9)	1	
	1–12 grade attended	59	5 (8.5)	1.94(0.54–6.97)	0.308
	Children	92	3 (3.3)	0.59(0.09–3.92)	0.585
Occupation	Pastoralist	497	38 (7.7)	1	
	Agro- pastoral	67	1 (1.5)	0.24(0.03–1.98)	0.187
	Others	66	7 (10.6)	1.34 (0.43–4.13)	0.616
Marital status	Married	464	38 (8.2)	1	
	Children	123	5 (4.1)	0.66(0.11–4.10)	0.657
	Other	43	3 (7.0)	1.10(0.29–4.18)	0.893
Body temperature	<37.5°c	545	41(7.5)	1	
	≥ 37.5°c	26	1 (3.9)	1.02(0.11–9.31)	0.985
Headache	No	155	13 (9.4)	1	
	Yes	473	32 (6.8)	0.99(0.45–2.17)	0.976
Joint pain	No	145	8 (5.5)	1	
	Yes	483	37 (7.7)	0.91(0.37–2.22)	0.839
Back pain	No	487	31(6.4)	1	
	Yes	141	15 (10.6)	1.39(0.66–2.94)	0.386
Illness duration	<8 days	239	10 (4.2)	1	
	9–30 days	158	14(8.9)	2.31(0.88–6.06)	0.090
	>30days	223	21(9.4)	2.70(1.02–7.18)	0.046

### Sero prevalence of *Rickettsia* infection

Of the 630 sera screened for *Rickettsia* infection by DCAT, 165 (26.2%) were reactive. Out of these sera, 41(21.8%), 33(17.6%), 9(4.8%) and 5(2.7%) were reactive at the titration of 1:80, 1:160, 1:320 and 1:640, respectively. Hence, 88 (53.3%) samples were reactive by the titration test at the cut off value ≥ 1:80. The combined sero-prevalence for *Rickettsia* infection by the two tests was 14.0% (88/630). The sero- prevalence was frequent among females compared to males (32.9% vs 15.8%, X^2^ = 22.74, P<0.001) by DCAT, as well as by titration test (18.5% vs. 6.9%, X^2^ = 16.83, P<0.001). It was also higher among those individuals who reported headache compared to who did not (28.3% vs 20.0%, X^2^ = 4.18, P = 0.041) by DCAT and (16.5% vs. 6.5%, X^2^ = 9.81, P = 0.002) by titration test. All the 25 samples which were non-reactive by DCAT were also non-reactive by titration test.

In multivariable regression analyses, being female (AOR = 3.10, 95%CI: 1.67–5.77, P <0.001) and reporting headache (AOR = 2.80, 95%CI: 1.26–6.22, P = 0.011) were significantly associated with sero-positivity for *Rickettsia* infection (Table 4).

**Table 4:** Socio-demographic of the study participants and sero-prevalence of typhus.

Characters		Number. tested	Number (%) positive by titration	AOR (95%CI)	P value
Sex	Male	247	17 (6.9)	1	
	Female	383	71 (18.5)	3.10 (1.67, 5.77)	<0.001
Age (years)	2–14	104	13 (12.6)	1	
	15–24	58	8 (13.8)	1.24 (0.29, 5.24)	0.768
	25–34	144	29 (20.1)	2.80 (0.66,11.87)	0.163
	35–44	117	13 (11.1)	1.26 (0.27,5.89)	0.768
	≥ 45	207	25 (12.1)	2.01 (0.46, 8.77)	0.352
Education	Illiterate	479	71 (14.8)	1	
	1–12 grade attended	59	7 (11.9)	1.16 (0.40,3.31)	0.785
	children	92	10 (11.0)	0.89 (0.29, 2.71)	0.839
Occupation	Pastoralist	497	77 (15.5)	1	
	agropastoral	67	5 (7.5)	0.51 (0.18,1.44)	0.204
	Others	66	6 (9.1)	0.60 (0.21,1.72)	0.344
Marital status	Married	464	67 (14.4)	1	
	Children	123	18 (14.7)	2.44 (0.75, 8.00)	0.140
	Other	43	3 (7.0)	0.44 (0.12,1.61)	0.217
Body temperature	<37.5°c	545	71 (13.1)	1	
	≥ 37.5° c	26	4 (15.4)	1.41 (0.41,4.88)	0.587
Headache	No	155	10 (6.5)	1	
	Yes	473	78 (16.5)	2.83 (1.26, 6.22)	0.011
Joint pain	No	145	15 (10.4)	1	
	Yes	483	73 (15.1)	1.52 (0.75,3.09)	0.241
Back pain	No	487	63 (13.0)	1	
	Yes	141	25 (17.7)	1.00 (0.52,1.94)	0.995
Illness duration	<8 days	239	31 (13.0)	1	
	9–30 days	158	29 (18.4)	1.38 (0.74,2.57)	0.312
	>30days	223	28 (12.6)	1.16 (0.57,2.38)	0.680

### Sero-prevalence of *Brucella* infection

The sero-prevalence for *Brucella* infection among the study participants was 12.7% (80/630) by RBPT and 35% (28/80) by CFT. The combined sero-prevalence for *Brucella* infection by the two tests was 4.4% (28/630). The sero-prevalence for *Brucella* infection was relatively high in the age group between 2–14 and 15–24 (Table 5). The sero-prevalence was also relatively high among individuals who reported drinking raw milk from aborted animals (13.0% vs. 6.9%) by RBPT and (20.6% vs 6.7%) by CFT. Among the study participants, 569 (90.7%) reported drinking raw milk from aborted animals, 566 (90.3%) touched aborted fetus/discharges from aborted animals without protection and 562 (90.1%) responded that they had no clear information about a disease that causes abortion in their animals.

**Table 5:** Socio-demographic characteristics of the study participants and prevalence of brucellosis.

Characters		Number tested	Number (%) positive by CFT	AOR (95% CI)	P value
Sex	Male	247	11(4.5)	1	
	Female	383	17 (4.4)	0.70(0.25–1.99)	0.509
Age (years)	2–14	104	9 (8.7)	1	
	15–24	58	4 (6.9)	0.25(0.03–2.07)	0.197
	25–34	144	5 (3.5)	0.29(0.02–3.43	0.323
	35–44	117	5 (4.3)	0.28(0.02–3.21)	0.310
	≥45	207	5 (2.4)	0.09(0.01–1.06	0.056
Education status	Illiterate	479	18 (3.8)	1	
	1–12 grade attended	59	3 (5.1)	0.38(0.06–2.45)	0.312
	Childern	92	7 (7.6)	0.23(0.03–1.63)	0.141
Occupation	Pastoral	497	18 (3.6)	1	
	agro pastoral	67	6 (8.9)	9.51(2.30–39.34)	0.002
	Others	66	4 (6.1)	3.43(0.55–21.50)	0.189
Marital status	Married	464	14 (3.0)	1	
	Children	123	12 (9.8)	4.15(0.62–27.66)	0.141
	Other	43	2 (4.7)	2.63 (0.46–4.88)	0.275
Drinking raw milk	No	58	1(1.7)	1	
	Yes	569	27 (4.7)	3.51(0.30–40.61)	0.315
Touching aborted materials	No	61	3 (4.9)	1	
	Yes	566	25 (4.4)	1.87 (0.28–12.26	0.514

In the univariable logistic regression analysis; being children (COR = 3.43,95%CI:1.40–8.40, P = 0.007) was found to be associated with high seropositivity for *Brucella* infection. On the other hand, age 45 and above was found to be associated with a low risk for *Brucella* infection (COR = 0.22, 95%CI: 0.06–0.75, P = 0.015). In multivariable logistic regression analysis, agropastoralism by occupation was associated with a high risk (AOR = 9.51, 95%CI: 2.30–39.34, P = 0.002) for *Brucella* infection. None of the 68 samples which were negative by RBPT was found positive by CFT.

The sero-prevalence of *Brucella* infection was not significantly associated with clinical symptoms reported by the study participants (Table 6).

**Table 6:** Sero-prevalence of *Brucella* infection and clinical features reported by the study participants.

Clinical Features		Test, RBPT		Test, CFT		P value
		Number tested	Number positive(%)	Number tested	Number positive(%)	
Body temperature	<37.5°c	545	65(11.9)	126	22(17.5)	0.960
	≥ 37.5° c	26	5(19.2)	6	1(16.7)	
Headache	No	155	15(9.7)	36	5(13.9)	0.376
	Yes	473	65(13.7)	112	23(20.5)	
Back pain	No	487	62(12.7)	114	24(21.1)	0.225
	Yes	141	18(12.8)	34	4(11.8)	
Malaise	No	469	57(12.2)	109	20(18.4)	0.767
	Yes	160	23 (14.4)	39	8(20.5)	
Joint pain	No	145	22(15.2)	37	8(21.6)	0.628
	Yes	483	58(12.0)	111	20(18.0)	
Weakness	No	539	65(12.1)	129	25(19.4)	0.709
	Yes	90	15(16.7)	19	3(15.8)	
Onset of the illness	<8 days	239	35(14.6)	63	13(20.6)	0.711
	9–30 days	158	19(12.0)	35	5(14.3)	
	>30 days	223	24(10.8)	48	8(16.7)	

### Prevalence of *P*. *falciparum* infection

Of the 650 suspected individuals for malaria, 16 (2.5%) were found positive for *P*. *falciparum* malaria infection microscopically, and *P*. *falciparum* was the only species detected.

*P*. *falciparum* malaria cases were more common among males than among females (4.4% vs 1.3%, X^2^ = 6.14, p = 0.013). The case was also high in the age group between 2–14 years (8.8%, X^2^ = 25.13, p < 0.001) and among individuals with body temperature ≥ 37.5°C (18.8% vs 1.6%, X^2^ = 35.80, p < 0.001). It was also high among individuals felt the illness for a week or less (4.4%, X^2^ = 6.59, p = 0.037). Multivariable regression analysis showed that being a male (AOR = 4.47, 95% CI:1.24–16.14, P = 0.022) and having fever ≥ 37.5 °C (AOR = 9.17, 95%CI: 1.96–42.84, P = 0.005) were independently associated with increased odds of having *P*. *falciparum* malaria infection (Table 7).

**Table 7:** Socio demographic of the study participants and prevalence of *P*. *falciparum* infection among the study participants.

Variables		Number diagnosed	Number (%) positive	AOR (95% CI)	P value
Sex	Female	397	5 (1.3)	1	
	Male	253	11(4.4)	4.47 (1.24–16.14)	0.022
Age (years)	2–14	114	10 (8.8)	1	
	15–24	59	2 (3.4)	0.04 (0.04–7.74)	0.649
	25–34	146	0 (0.0)	Empty	
	35–44	120	2 (1.7)	0.63 (0.03–11.67)	0.755
	≥ 45	211	2 (1.0)	0.25 (0.01–5.41)	0.377
Occupation	Pastoralist	514	13 (2.5)	1	
	Agro pastoral	67	1 (1.5)	4.76 (0.38–16.15)	0.227
	Others	69	2 (2.9)	1.98 (0.28–14.24)	0.496
Education	Illiterate	489	6 (1.2)	1	
	1–12 grade attended	61	2 (3.3)	0.40 (0.04–4.23)	0.444
	Children	100	8 (8.0)	1.71 (0.27–10.74)	0.569
Marital status	Married	474	4 (0.8)	1	
	Children	133	11(8.3)	2.34(0.21–25.84)	0.488
	Other	43	1(2.3)	1.68 (0.12–24.16)	0.700
Body temperature	<37.5°c	557	9 (1.6)	1	
	≥ 37.5° c	32	6 (18.8)	9.17 (1.96–42.84)	0.005
Headache	No	160	2 (1.3)	1	
	Yes	486	14 (2.9)	3.64 (0.64–20.80)	0.146
Joint pain	No	160	7 (4.4)	1	
	Yes	486	9 (1.9)	2.04 (0.44–9.40)	0.360
Onset of the illness	<8 days	249	11(4.4)	1	
	9–30 days	161	1(0.6)	0.21(0.02–1.92)	0.167
	>30days	228	4 (1.8)	2.25 (0.47–10.80)	0.312

### Concurrent infections

Among the total 650 study participants who were tested for *S*. Typhi, *Rickettsial*, *Brucella* and/or *Plasmodium* infections, 344 (52.9%) were found to be positive for one or more of the infectious agents by the screening tests (Widal and Weilfelix direct card agglutination, Rose Bengal Plate Test and blood films). However, only 24.6% (160/650) were found to be positive for one or more of the infectious agents by the confirmatory tests (titration test for *S*. Typhi *and Rickettsia* infections, and Complement Fixation Test for *Brucella* infection).

## Discussion

We investigated the prevalence of typhoid fever, typhus, brucellosis and malaria among individuals reported signs of fever, headache, joint pain and back pain in Amibara district, Afar Region, Ethiopia, through a community-based cross-sectional study. A quarter of the individuals (24.6%) were sero-positive for *S*.Typhi, *Rickettsia*, *Brucella* infection by confirmatory tests, and/or positive for *P*. *falciparum* infection by microscopy. This result is in line with previous health facility based studies in other parts of Ethiopia on causes of febrile illnesses [18, 19], and the highest disease prevalence was found for typhus (14.0%) followed by typhoid fever (7.3%).

However, the sero-prevalence of *Rickettsia* infection in this study was lower than the one reported from other parts of Ethiopia [19, 28], but higher than the findings of the studies by Tadesse and Tadesse [18] and Birhane et al. [29]. The variation might be linked to the type of environment in the study area. Studies also showed that the occurrence of typhus in Ethiopia is linked to poor hygienic/crowded living condition, where it can cause high mortality rates [30]. The sero-prevalence of *Rickettsia* infection was high among individuals reported headache. Headache has been shown to be one of the main clinical symptoms of endemic typhus [31]. The study also showed that the seroprevalence for *Rickettsia* infection was more common among females than among males. This high sero-prevalence for *Rickettsia* infection among females could be due to the large number of female study participants involved in this study. A previous retrospective sero-prevalence study of typhus among prisoners in Ethiopia also showed a higher seroprevalence among males than among females which could be due to a higher proportion of male study participants (86%) compared to that of female study participants (14%) [28]. However, further well designed community-based study is needed to investigate the reason including differences in treatment-seeking behaviour among adult females and males in the present pastoral area.

Nevertheless, our study showed that typhus is one of the major public health concerns in the study area. Hence, emphasis should be given to appropriate diagnosis/treatment and prevention of *Rickettsia* infection like through increasing community’s awareness in the present study area.

The second most common disease found was typhoid fever (7.3%). Our result is comparable with other health facility based studies in different parts of Ethiopia [18, 24]. The observed sero-prevalence is lower than the results of health facility based studies in Ziway area [19] and Northwest Ethiopia [29]. Various factors could explain these differences: seasons, environmental hygiene, geographical location and the nature of the study population [32]. The study also showed that the seroprevalence for *S*.Typhi infection was slightly high among females which is similar to the results of other previous study in Ethiopia [18]. In the present study, the proportion (61%) of female participants was higher than the proportion (39%) of male participants, and this might contribute to the observed high sero prevalence for *S*.Typhi infection among females. However, a previous health facility-based study in Ethiopia revealed a slightly higher seroprevalence for *S*.Typhi infection among female study participants (22.5%) compared to that of males (16.7%) despite a higher proportion of male participants (60%) compared to that of females (40%) [29]. In present study area, pastoralists share stagnant and open natural water sources with their livestock, which increases the risk for getting *S*.Typhi infection and favors the spread of the disease. Moreover, the high seroprevalence for *S*.Typhi infection among females could be associated with the daily living habits of females like frequency of exposure to contaminated water during fetching water from river and wells or washing clothes as most of these activities are usually performed by females.

In addition, health facilities in the study area had limited laboratory facilities to accurately diagnosis and treat the disease. Thus, increasing access to safe water, strengthening health facility/system for the diagnosis of typhoid fever and treatment as well as increasing community awareness are very important in order to reduce the mortality and morbidity due to this disease.

Several studies have shown the occurrence of brucellosis in livestock in different parts of Ethiopia [33–35]. However, there was no health facilities/community based information on the status of brucellosis in humans in the present study area. The overall sero-prevalence of brucellosis in the study participants was 4.4% by RBPT and CFT. The result is higher than the finding of health facilities based previous studies in individuals with febrile illness in other part of Ethiopia [19,36], but lower than the results of health facility based study from Borena area, South Ethiopia and Metema area, north Ethiopia [17]. Another recent study from Jimma area (south Ethiopia) also revealed a low sero-prevalence of *Brucella* infection as detected by RBPT (2.1%) and CFT (0.0%) [37].

Brucellosis has an overlap of clinical symptoms with many other febrile diseases, and can be misdiagnosed with malaria or other diseases due to lack of awareness of medical staff and lack of diagnostic capabilities in the present study area. In this study, 4.4% of symptomatic study participants who were found sero-positive for *Brucella* infection, would not have been diagnosed for brucellosis if the physician would have based the diagnosis solely on clinical signs. Therefore, efforts need to be made to improving laboratory services for the diagnosis of brucellosis in the present study area.

In the present study, significant difference was not found in the prevalence of brucellosis between males and females, which is similar with other studies done in Ethiopia [36], Tanzania [38] and Kenya [39]. However, a hospital based study in Uganda showed a higher sero-prevalence of *Brucella* infection among males than in females [40]. This can be explained by the fact that among pastoralists, both women and men are equally exposed to risk factors for *Brucella* infection. Unlike results from Uganda [40] and Bangladesh [41], where elders were more affected by brucellosis, our study showed relatively high prevalence of *Brucella* infection in the younger age group and children. Children can be exposed to *Brucella* infection by regularly drinking raw milk, contaminated soil with the bacteria and having regular close contact with livestock, particularly goat and sheep [35].

This study, was undertaken during a high malaria season though it showed a low prevalence of undiagnosed and untreated malaria (2.5%) among the study participants compared to results of previous community-based prevalence study of malaria among non-febrile individuals in Gondar town, North Ethiopia [42], and in the pastoral community of the Bena-Tsemay district, South Ethiopia [43]. Although this low prevalence of malaria might be due to the result of the prevention and control measures employed by the Ministry of Health to eliminate malaria in the country [44], the present observed prevalence of undiagnosed and untreated malaria cases should not be considered insignificant since these undiagnosed and untreated individuals would contribute to the transmission of the disease among the community. Moreover, in the present study area, *P*. *falciparum* which causes the most severe form of malaria is the widely distributed species as previously reported [45]. Hence, strengthening community based malaria case detection in this area, for example through community health extension workers is very important in order to achieve the plan for malaria elimination.

In this study, among other clinical features, body temperature ≥ 37.5 °C was found to be strong indicator for infection with *falciparum* malaria. Previous studies also suggested that increased body temperature could be helpful in diagnosing and treating children with febrile illness [46, 47].

In Ethiopia, serological tests (Widal and Weil-Felix) using the DCAT are commonly used to diagnose typhoid fever and typhus. A number of subsequent studies indicated that these tests are extremely valuable in the absence of adequate laboratory facilities and culture methods like in resources limited countries [48–51].

On the other hand, several previous studies have shown a high seroprevalence of *S*.Typhi infection using serological based screened tests, but revealed absence or very low prevalence using blood culture or fecal samples which are considered as gold standard tests for the diagnosis of a current infection with *S*.Typhi [24, 29, 52, 53]. Bacteriological isolation is the gold standard for the diagnosis of current infection with *Brucella*. RBPT was found to be simple and useful for the screening for *Brucella* infection in health institutions where bacterial culturing facilities are not available [25], though it is not a useful test to distinguish between acute and chronic *Brucella* infection [54]. Whereas, CFT can be used as a confirmatory test.

In this study, we have used Widal, Weil-Felix, and RBPT/CFT tests to report the seroprevalence for *S*.Typhi, *Rickettsia and Brucella* infections, respectively despite the fact that these serological based tests are not convincing tests for the diagnosis of current infection because of short comings such as false positivity due to previous exposure or false negativity in an endemic setting [24], and this could be one of the major limitations of the present study.

The study participants were recruited based on the clinical signs/symptoms reported by the study participants that may not necessarily have been caused by an infectious agents that cause acute or chronic illness and this might result in a selection bias. The primary objective of this study was to identify the prevalence of brucellosis, malaria, typhoid and typhus among symptomatic individuals with febrile illness related symptoms. However, in an endemic area, these diseases could be prevalent among asymptomatic individuals. Hence, the findings of this study cannot be generalized to the entire population in the study area.

In pastoralists like in the present study area who are living in close contact with their animals on a daily basis, many neglected zoonotic diseases such as campylobacteriosis, Q fever, and leptospirosis can cause a significant health problems both in humans and animals [55]. In the present study, among 650 individuals who complained various illnesses, only 160 (24.6%) were diagnosed for one or more of the above mentioned diseases, and in the majority (75.4%) of the symptomatic individuals the cause of their illness remained unknown, because diagnosis of other related diseases was not considered due to lack of diagnostic tools/reagents and this could also be considered as one of the limitations of this study.

### Conclusion

In this study, typhoid fever, typhus, brucellosis and malaria were observed among symptomatic individuals. The study also highlighted that brucellosis cases can be misdiagnosed as malaria or other disease based solely on clinical diagnosis. Therefore, efforts are needed to improve disease awareness and laboratory services for the diagnosis of brucellosis, which should be considered in the routine differential clinical diagnosis of febrile illness in the study area. Only a quarter of the study participants (24.6%) were diagnosed for one or more of the above mentioned diseases. In the majority (75.4%) of the symptomatic individuals, the cause of their illness remained unknown. In addition, a high prevalence of unexplained abortions in women (35.7%) was observed. Hence, further community based studies on other zoonotic diseases like leptospirosis and Q fever are warranted to identify other causes of febrile illness in this pastoral setting.

## Supporting information

S1 Checklist STROBE checklist. (DOC)
