# Supplementary material for: Community-based prevalence of typhoid fever, typhus, brucellosis and malaria among symptomatic individuals in Afar Region, Ethiopia
Source: PLoS Negl Trop Dis. 2018 Oct 4;12(10):e0006749. doi: 10.1371/journal.pntd.0006749 (PMC6191156; doi:10.1371/journal.pntd.0006749)
Supplement: S1 Checklist — (DOC) [file pntd.0006749.s001.doc]

STROBE Statement—Checklist of items that should be included in reports of ***cross-sectional studies***

|  | Item No | Recommendation |
| --- | --- | --- |
| **Title and abstract** | 1 | (*a*) Indicate the study’s design with a commonly used term in the title or the abstract  Fulfilled |
|  |  | (*b*) Provide in the abstract an informative and balanced summary of what was done and what was found Indicated in the abstract |
| Introduction | | |
| Background/rationale | 2 | Explain the scientific background and rationale for the investigation being reported Indicated in the introduction part |
| Objectives | 3 | State specific objectives, including any prespecified hypotheses Indicated in the introduction section , |
| Methods | | |
| Study design | 4 | Present key elements of study design early in the paper Indicated in the method section |
| Setting | 5 | Describe the setting, locations, and relevant dates, including periods of recruitment, exposure, follow-up, and data collection Indicated in the method section |
| Participants | 6 | (*a*) Give the eligibility criteria, and the sources and methods of selection of participants Indicated in the method section |
| Variables | 7 | Clearly define all outcomes, exposures, predictors, potential confounders, and effect modifiers. Give diagnostic criteria, if applicable Indicated in the method section under **Data management and** **analysis** |
| Data sources/ measurement | 8* | For each variable of interest, give sources of data and details of methods of assessment (measurement). Describe comparability of assessment methods if there is more than one group Indicated in the method section under **Data management and** **analysis** |
| Bias | 9 | Describe any efforts to address potential sources of bias |
| Study size | 10 | Explain how the study size was arrived at Indicated in the method section under **Study design, sample size estimation and data collection** |
| Quantitative variables | 11 | Explain how quantitative variables were handled in the analyses. If applicable, describe which groupings were chosen and why, Indicated in the method section under **Data management and** **analysis** |
| Statistical methods | 12 | (*a*) Describe all statistical methods, including those used to control for confounding Indicated in the method section under **Data management and** **analysis** |
|  |  | (*b*) Describe any methods used to examine subgroups and interactions |
|  |  | (*c*) Explain how missing data were addressed |
|  |  | (*d*) If applicable, describe analytical methods taking account of sampling strategy |
|  |  | (*e*) Describe any sensitivity analyses |
| Results | | |
| Participants | 13* | (a) Report numbers of individuals at each stage of study—eg numbers potentially eligible, examined for eligibility, confirmed eligible, included in the study, completing follow-up, and analysed Indicated in the result section under **Socio-demographic characteristics of the study participants , paragraph 1** |
|  |  | (b) Give reasons for non-participation at each stage |
|  |  | (c) Consider use of a flow diagram |
| Descriptive data | 14* | (a) Give characteristics of study participants (eg demographic, clinical, social) and information on exposures and potential confounders Indicated (Table 1) |
|  |  | (b) Indicate number of participants with missing data for each variable of interest |
| Outcome data | 15* | Report numbers of outcome events or summary measures Reported in T able 2-6 |
| Main results | 16 | (*a*) Give unadjusted estimates and, if applicable, confounder-adjusted estimates and their precision (eg, 95% confidence interval). Make clear which confounders were adjusted for and why they were included Indicated Table 6 |
|  |  | (*b*) Report category boundaries when continuous variables were categorized Table 2-6 |
|  |  | (*c*) If relevant, consider translating estimates of relative risk into absolute risk for a meaningful time period |
| Other analyses | 17 | Report other analyses done—eg analyses of subgroups and interactions, and sensitivity analyses |
| Discussion | | |
| Key results | 18 | Summarise key results with reference to study objectives indicated in the discussion part |
| Limitations | 19 | Discuss limitations of the study, taking into account sources of potential bias or imprecision. Discuss both direction and magnitude of any potential bias indicated in the discussion part |
| Interpretation | 20 | Give a cautious overall interpretation of results considering objectives, limitations, multiplicity of analyses, results from similar studies, and other relevant evidence indicated in the discussion part |
| Generalisability | 21 | Discuss the generalisability (external validity) of the study results indicated in the discussion part |
| Other information | | |
| Funding | 22 | Give the source of funding and the role of the funders for the present study and, if applicable, for the original study on which the present article is based  The study was financially supported by This Study was financially suported by the Institute of Tropical Medicine (ITM-Belgium).  The funding agency had no any role in conducting the study and in preparing the manuscript. |

*Give information separately for exposed and unexposed groups.

**Note:** An Explanation and Elaboration article discusses each checklist item and gives methodological background and published examples of transparent reporting. The STROBE checklist is best used in conjunction with this article (freely available on the Web sites of PLoS Medicine at http://www.plosmedicine.org/, Annals of Internal Medicine at http://www.annals.org/, and Epidemiology at http://www.epidem.com/). Information on the STROBE Initiative is available at www.strobe-statement.org.
